# Fifty Shades of E^rns^: Innate Immune Evasion by the Viral Endonucleases of All Pestivirus Species

**DOI:** 10.3390/v14020265

**Published:** 2022-01-27

**Authors:** Elena de Martin, Matthias Schweizer

**Affiliations:** 1 Institute of Virology and Immunology, Länggass-Str. 122, POB, CH-3001 Bern, Switzerland; elena.demartin@vetsuisse.unibe.ch; 2 Department of Infectious Diseases and Pathobiology, Vetsuisse Faculty, University of Bern, CH-3012 Bern, Switzerland; 3 Graduate School for Cellular and Biomedical Sciences, University of Bern, CH-3012 Bern, Switzerland

**Keywords:** pestivirus, bovine viral diarrhea virus (BVDV), viral endonuclease, innate immune evasion, interferon type-I, atypical pestiviruses, viral RNase

## Abstract

The genus *Pestivirus*, family *Flaviviridae*, includes four historically accepted species, i.e., bovine viral diarrhea virus (BVDV)-1 and -2, classical swine fever virus (CSFV), and border disease virus (BDV). A large number of new pestivirus species were identified in recent years. A common feature of most members is the presence of two unique proteins, N^pro^ and E^rns^, that pestiviruses evolved to regulate the host’s innate immune response. In addition to its function as a structural envelope glycoprotein, E^rns^ is also released in the extracellular space, where it is endocytosed by neighboring cells. As an endoribonuclease, E^rns^ is able to cleave viral ss- and dsRNAs, thus preventing the stimulation of the host’s interferon (IFN) response. Here, we characterize the basic features of soluble E^rns^ of a large variety of classified and unassigned pestiviruses that have not yet been described. Its ability to form homodimers, its RNase activity, and the ability to inhibit dsRNA-induced IFN synthesis were investigated. Overall, we found large differences between the various E^rns^ proteins that cannot be predicted solely based on their primary amino acid sequences, and that might be the consequence of different virus-host co-evolution histories. This provides valuable information to delineate the structure-function relationship of pestiviral endoribonucleases.

## 1. Introduction

The genus *Pestivirus* belongs to the family *Flaviviridae,* and includes pathogens of primary economic importance in livestock. The most impactful species are bovine viral diarrhea virus 1 and 2 (BVDV-1 and BVDV-2), and classical swine fever virus (CSFV), both included in the World Organization for Animal Health (OIE) list of notifiable diseases 2021. In addition, there are several related viruses that have been officially recognized, making the establishment of a new taxonomy for pestiviruses necessary. Such new taxonomy is substituting the generic names with Latin letters (e.g., *Pestivirus A* for BVDV-1), with a total of 11 recognized and some unassigned pestivirus species [1,2].

For successful infection, pestiviruses encode for interferon (IFN) antagonists to prevent the activation of the innate immune system. Thus, most pestiviruses evolved two proteins that are unique to the genus, the N-terminal non-structural autoprotease N^pro^, and the envelope glycoprotein and endoribonuclease E^rns^. Both proteins have been shown to be required for a complementary, non-redundant, inhibition of the interferon (IFN) cascade upon double-stranded (ds) RNA stimulation [3,4] (for reviews, see [5,6,7]). In particular, E^rns^ is both, a structural and a non-structural component of the virus (“E” for envelope glycoprotein and “rns” for RNase secreted), with the latter also being released in a soluble form in the extracellular space and taken up by neighboring cells by endocytosis [8,9]. The most studied E^rns^ proteins are those from CSFV and BVDV, and it was shown that this peculiar RNase is able to cleave viral single- (ss) and dsRNAs [10,11,12,13], thus preventing the stimulation of the innate immune response by such immunostimulatory pathogen-associated molecular patterns (PAMPs) in a dose-dependent fashion [4,9,12,14]. Structurally, BVDV-E^rns^ is a protein of 227 amino acids, with an RNase composed by two domains within its N-terminus that correspond to nucleases of the RNase T2 family members [10,15]. At its C-terminus, the protein harbors an amphipathic helix that functions as membrane anchor, where it is embedded in plane and facilitates its release into the extracellular matrix [16,17,18,19]. The protein of BVDV has nine cysteine residues (C), with the first eight being engaged in intramolecular disulfide bridges, whereas the last one is engaged in an intermolecular disulfide bridge forming E^rns^ homodimers [20]. In addition, the C-terminal end of E^rns^ contains a positively charged domain that enables the protein to interact with cell surface glycosaminoglycans (GAG) [21,22,23]. The presence of this GAG-binding site is required for cell attachment followed by its uptake via clathrin-mediated endocytosis [9], leading most likely to the localization of E^rns^ in an endolysosomal compartment [7,24].

Knowledge concerning members of the *Pestivirus* genus has been expanding in recent years, with more and more viruses being discovered and identified. However, besides the well-known species BVDV-1, -2 and CSFV (*Pestivirus A*, *B*, and *C*, respectively), the basic features of the E^rns^ proteins of the “non-classical” pestiviruses have not yet been characterized. With E^rns^ being a key element for the interaction between pestiviruses and the host’s innate immune system, we considered the study of E^rns^ as a pivotal requisite to understand the biology of these viruses, their evolutionary strategies, and the possible containment measures against them. Thus, we aim to provide a more complete picture on the main functions of E^rns^ among different pestiviruses, characterizing the E^rns^ proteins from the less studied pestiviruses. The assigned and proposed species analyzed in this study are summarized in Table 1. We investigated the ability of E^rns^ to dimerize, its enzymatic activity on dsRNA in vitro, the potential to inhibit dsRNA-induced IFN synthesis, and the intracellular localization of E^rns^ as the main features of this pestiviral IFN antagonist to inhibit their host’s innate antiviral response. In addition, to further define structure-function relationships, we constructed several mutants of E^rns^ proteins that allowed us to unveil the dichotomous effect of a key point mutation on the RNase activity of E^rns^.

## 2. Materials and Methods

### 2.1. Cells and Reagents

Primary bovine turbinate (BT) cells were generated from fetuses obtained from local abattoirs, processed in our institute, and tested negative for BVDV by immunoperoxidase staining. During experiments, cells were maintained in a humidified atmosphere with 5% CO_2_ at 37 °C, with Eagle’s minimal essential medium (MEM) containing 7% fetal bovine serum (FBS). FBS was provided by PAA laboratories (Lucerna-Chem AG, Lucerne, Switzerland), and was free of pestivirus and antibodies to BVDV/BDV as tested by virus isolation and SNT, respectively [40]. HEK and CHO cells were used for protein expression by the “Protein Production and Structure Core Facility” of the Swiss Federal Institute of Technology (EPFL) in Lausanne, Switzerland.

### 2.2. Expression and Purification of Strep-Tagged E^rns^

**Figure 1:**
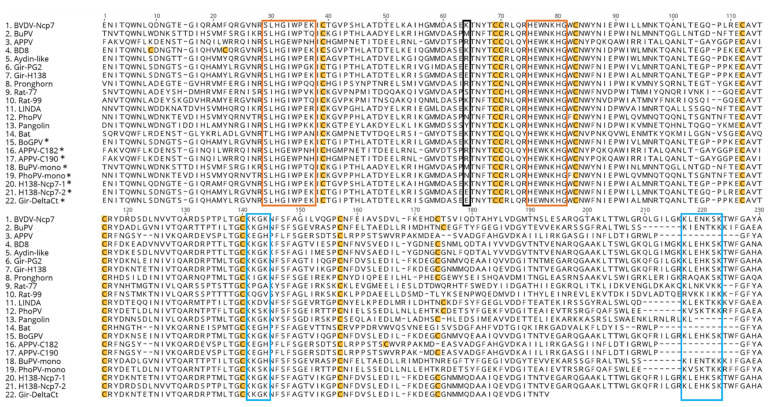
Amino acid alignment of the sequences of the E^rns^ analyzed in this study. Sequences carrying the asterisk were not published elsewhere. The corresponding references and, where available, the GenBank numbers are reported in Table 1. To prevent the cleavage of the Strep-tag, the last four amino acids at the C-terminus of APPV-, Bat-, Rat77-, Rat99- and Pangolin-E^rns^, were modified in “GFYA”. In yellow are highlighted the cysteine (C) residues important for intra- and inter-molecular disulfide bridges. The orange boxes are focusing on the two RNase active sites conserved among most pestiviruses (except for APPV- and Bat-pestivirus), whereas the black box highlights the residue important for RNase activity as identified in this study using E^rns^ of giraffe pestiviruses. The proposed “positive-region” and “heparin-binding-domain” [24] are boxed in blue.

The list of E^rns^ sequences used in this study is summarized in Table 1, with the species annotation according to the established and proposed nomenclature [1,25]. The phylogenetic relationship of the various pestivirus species is described in [41]. For some proteins, e.g., APPV E^rns^, the last four amino acids were replaced by a GFYA sequence to avoid cleavage of the Strep-tag domain by the signal peptidase [42,43]. The sequences of the various E^rns^ proteins used in this study with the respective modifications are shown in Figure 1 and summarized in Table 1.

A Twin-Strep-tag (WSHPQFEKGGGSGGGSGGSAWSHPQFEK) sequence was added at the C-terminus, separated from the main sequence by a linker (SA), and a mouse IgG kappa light chain signal sequence (METDTLLLWVLLLWVPGSTG) was located at the N-terminus, as previously described [44]. After codon optimization, we added a Kozak sequence (GCCACC) to the N-terminal end of the construct, yielding a final structure of “Kozak–kappa–linker–E^rns–^Strep-Tag”. The complete DNA sequences were synthesized by Twist Bioscience (San Francisco, CA-USA). Each DNA sequence was ligated into a pCI mammalian expression vector (Promega; kindly provided by P. Plattet, Neurovirology, University of Bern) using the “In-Fusion HD cloning kit” from Clontech (Takara Bio Company, Saint-Germain-en-Laye, France), and sequences were confirmed by Sanger sequencing. Primers and sequencing were produced and performed by Microsynth (Balgach, Switzerland). The plasmids obtained were used to transfect HEK293 cells, cultivated in suspension at the “Protein Production and Structure Core Facility” (EPFL, Lausanne, Switzerland) for seven days in Freestyle 293 medium (GIBCO Life Technology; Thermo Fisher Scientific (Schweiz) AG, Reinach, Switzerland) supplemented with valproic acid [45]. Each supernatant of the transfected cells containing the secreted E^rns^ was purified as described [44] through a dedicated gravity flow Strep-Tactin XT Superflow column (IBA GmbH, Göttingen, Germany), following the manufacturer’s protocol. After purification, the concentration of each product was quantified with a NanoDrop spectrometer (Thermo Scientific; Witec AG, Sursee, Switzerland) with A280 measurements.

To exclude a possible effect of the cell culture system used in case we experienced no E^rns^ expression (compare Section 3.1) or no RNase activity (see Section 3.2), we transfected HEK cells with and without the addition of valproic acid [45] and CHO cells with and without DMSO [46]. However, even though the pattern of protein glycosylation is vastly different between HEK and CHO cells [47], we did not expect glycosylation to be the cause, as E^rns^ expressed in insect, bovine, and human cells show comparable activity as RNase and IFN antagonist [4,44]. The resulting supernatants were directly analyzed by Western blot targeting the Strep-tag domain without prior purification, but none of the preparations gave a different result compared to the standard method.

### 2.3. RNase Activity Assay

A stretch of 300 bp of dsRNA was obtained from the 5′-UTR of the BVDV strain Ncp7 genome, as previously described [9,14]. The various E^rns^ proteins were diluted in elution buffer (IBA GmbH) at pH 7, and the dsRNA substrate in 100 mM Tris/acetate at pH 5.5 to yield a concentration of 50 ng/µL. Equal volumes of E^rns^ and dsRNA were mixed and incubated at 37 °C for 60 min for enzymatic digestion. After cooling, 2 × RNA loading dye (NEB; Bioconcept, Allschwil, Switzerland) was added to each reaction, and samples were run on a 1% agarose gel with ethidium bromide at 100 V for 45 min. Gels were visualized by UV light using an E-Box CCD camera (Vilber; Witec AG). To quantify the enzymatic activity on dsRNA, we analyzed the agarose gel images with the Vilber Fusion^©^ Software (Witec AG), quantifying the signal of RNA residues at the height of 300 bp. Data interpolation was performed to estimate the amount of each E^rns^ required to reduce the signal by 50%.

### 2.4. Mx Assay

BT cells were seeded in 12 well plates and treated after adherence of the cells. Treatment consisted in 500 µL of MEM with 2% FBS containing fixed concentrations of E^rns^ at 50, 25, 20, 10, 5, and 1 ng/µL). After 30 min of incubation at 37 °C, cells were washed twice with 0.1 mg/mL heparin sulfate to remove soluble extracellular proteins and E^rns^ attached to the cell surface. Thereafter, cells were incubated with 2 µg/mL of poly(IC) for 22 h, in order to induce the expression of the interferon-induced GTP-binding protein Mx that we used as a proxy to evaluate the expression of interferon [48]. After incubation, adherent cells were lysed with 60 µL M-Per mammalian protein extraction reagent (Pierce; Thermo Fisher Scientific (Schweiz) AG) containing complete protease inhibitor (Roche Diagnostics, Rotkreuz, Switzerland), and stored at −20 °C until analysis by Western blot.

### 2.5. Western Blot

Cell lysates (12 µL) were mixed with 3 µL of Lämmli buffer containing 2% β-mercaptoethanol (2-ME) and boiled at 95°C for 5 min After cooling, samples were loaded on precast polyacrylamide SurePAGE^TM^ Bis-Tris 8% gels (GenScript; Witec AG) and run for 45 min at 180 V in MOPS buffer. As chemiluminescence substrate, WesternBright ECL HRP substrate (advansta Inc. USA; Witec AG) was used. Electroblotting onto nitrocellulose membranes (Amersham Bioscience, Switzerland) was achieved with eBlot^TM^ L1 Fast Wet Transfer System (GenScript; Witec AG). After transfer, nonspecific binding was prevented by membrane blocking with 5% low-fat dry milk for 1 h at room temperature. Images were detected and analyzed by a Fusion FX7 CCD camera (Vilber; Witec AG).

For staining of cellular Mx proteins, a primary mouse monoclonal antibody against MxA was used [24]. As a house-keeping protein, β-actin was detected with a mouse monoclonal anti-β-actin antibody (Sigma, Buchs, Switzerland). As a secondary antibody, peroxidase-conjugated AffiniPure Donkey anti-mouse IgG (Jackson ImmunoResearch Europe Ltd.; Milan Analytica AG, Rheinfelden, Switzerland) was used to reveal both primary antibodies.

### 2.6. Coomassie Staining

To evaluate the molecular weight of the various E^rns^ proteins, 2 μg of each were analyzed under non-reducing conditions as previously described [24]. Briefly, the proteins were separated by sodium dodecyl sulfate-polyacrylamide gel electrophoresis (SDS-PAGE) followed by standard staining with Coomassie Brilliant Blue G250 (Bio-Rad Laboratories, Cressier, Switzerland).

### 2.7. Immunofluorescence Microscopy

For immunofluorescence analyses, BT cells were grown on cover glasses with thickness No. 1.5H (Paul Marienfeld GmbH & Co. KG, Lauda-Königshofen, Germany) in a 24 well-plate. After adherence, cells were incubated with 10 ng/µL of each E^rns^ protein for 30 min at 37 °C, diluted in MEM containing 2% FBS. After incubation, cells were washed twice with 0.1 mg/mL heparin sulfate and fixed with 4% formalin for 15 min. The indirect labeling was performed using a monoclonal mouse anti Strep-tag antibody (IBA) as previously described [24]. Visualization was performed using an EVOS^TM^-FL Auto 2 imaging system (Invitrogen, Thermo Fisher Scientific) with a 40× air objective. Image processing was done with Fiji (ImageJ) [49].

## 3. Results

### 3.1. Protein Expression and Molecular Weight Determination

The E^rns^ proteins from the various pestivirus species were purified from the supernatant of transiently transfected HEK cells, and their purity was verified by SDS-PAGE and Coomassie staining as described in the Methods section. Exceptionally, the wild-type (wt) form of APPV-E^rns^ could not be expressed or purified by the Strep-tag affinity purification. As the nucleotide sequence of APPV-E^rns^, including its C-terminal end, is rather different from BVDV, the Strep-tag at the C-terminal end might have been cleaved by the signal peptidase, which possess rather complex sequence requirements for substrate recognition [42,43,50]. Thus, we expressed a chimeric protein of APPV-E^rns^ substituting its last 24 amino acids with the last 39 amino acids of BVDV-Ncp7-E^rns^, which indeed allowed us to successfully express and purify the chimeric protein. As the last four amino acids (VAEA) might act as a von Heijne motif [51] leading to the cleavage of the Strep-tag sequence, we narrowed the mutations down to this region of the sequence. Thus, replacing the last four amino acids (VAEA) of our APPV strain with GFYA prevented proteolytic cleavage of the tag and allowed successful purification of APPV-E^rns^. Therefore, we preventively applied the same mutations to the E^rns^ proteins of Bat, Rat-77, Rat-99, and Pangolin pestiviruses.

**Figure 2:**
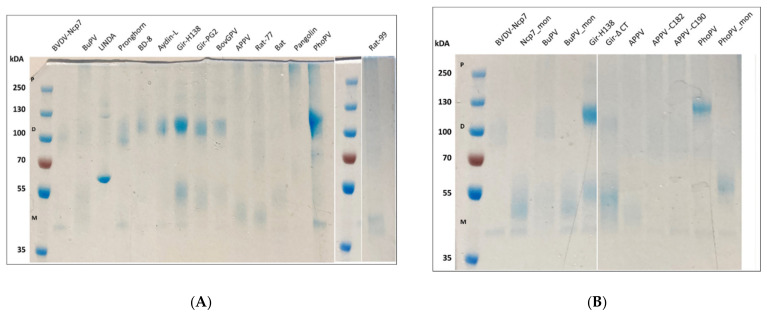
Coomassie staining of the various E^rns^ proteins analyzed. (**A**) Profile of the wild-type (wt) proteins. The absence of a cysteine in a position analogous to BVDV-Ncp7-171 is reflected in the absence of dimer formation (APPV-, Rat-77-, Rat-99-, and Bat-pestiviruses). (**B**) Profile of wt and mutant E^rns^ proteins. Mutant proteins were prevented from dimerization by mutating the corresponding cysteine residue into arginine (Ncp7-mon, BuPV-mon, and PhoPV-mon). The introduction of a ninth cysteine residue on either position 182 or 190 of APPV-E^rns^ did not induce an appreciable dimerization. Next to the pre-stained size ladder, approximate heights of monomers (M), dimers (D), and polymers (P) are indicated. Uncropped gels are available in Supplementary Material Figure S1.

To obtain information about the quaternary structure of the different E^rns^ proteins, we separated each purified protein by SDS-PAGE under non-reducing conditions and stained it with Coomassie Brilliant Blue (Figure 2A,B). The results are consistent with the predictions based on the primary structure of the proteins, i.e., in the absence of the cysteine at position 171 or a cysteine in analogous position (Figure 1; numbering according to E^rns^ of strain BVDV-Ncp7 [44]), only the monomeric form is detected, whereas in the presence of this residue, monomeric, dimeric, and polymeric forms could be identified. Specifically, APPV, Bat- and both Rat-E^rns^ proteins were only present in form of monomers and polymers, but not dimers, and the staining intensity in the gel appeared to be lower than most of the other proteins. Additionally, the presence of two additional cysteines in the N-terminus of BD8-E^rns^ did not appear to play a role, neither in dimerization of the protein nor for its RNase activity compared to similar proteins without these additional cysteines (e.g., Aydin-like-E^rns^). Consistently, artificial mutants of BVDV-Ncp7-, BuPV- and PhoPV-E^rns^, where the ninth cysteine was substituted by an arginine, could not form dimers (Figure 2B). On the other hand, the insertion of a cysteine residue in either position 182 or 190 in the APPV-E^rns^ did not induce a change of pattern appreciable by Coomassie staining (Figure 2B).

### 3.2. RNase Activity

**Figure 3:**
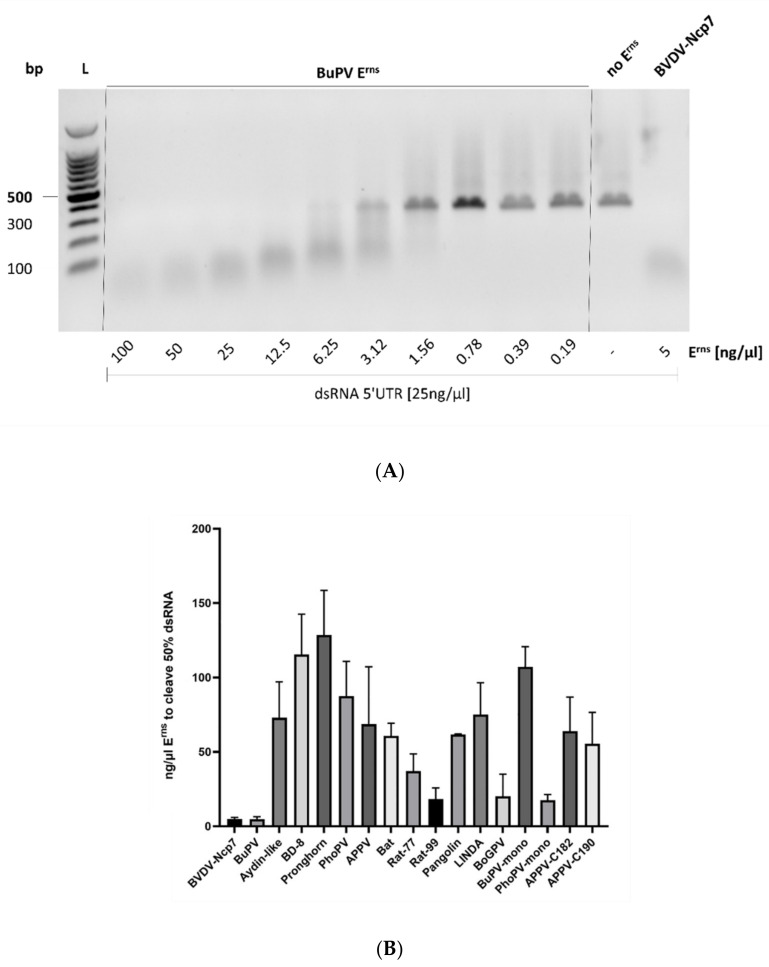
RNase activity of the E^rns^ proteins of different pestivirus species analyzed. (**A**) Agarose gel of BuPV-E^rns^ is shown as an example for the enzymatic assay, with decreasing signal for the 300 bp dsRNA with increasing concentrations of E^rns^. The sizes of the 100, 300, and 500 bp fragments within the 100 bp ladder (L) as size marker are indicated. The uncropped gel is available in Supplementary Material Figure S2. (**B**) Quantification of the gels as shown in panel A, estimating the amount of the various E^rns^ proteins required to reduce the signal of dsRNA by 50% (n = 3 ± SD). Proteins that did not display any detectable activity (i.e., E^rns^ of Gir-PG2, Gir-H138 and Gir-ΔCt), or for which the 50% value could not be calculated (i.e., the chimeric E^rns^ proteins H138-Ncp7-1 and H138-Ncp7-2), are not shown. The activities relative to E^rns^ of BVDV-Ncp7 are shown in Table 1.

The RNase activity of the various E^rns^ proteins from the different pestivirus species was analyzed by an RNase activity assay with dsRNA as substrate [14,44], as described in the Methods section. The analysis with E^rns^ of Bungowannah virus is shown as an example (Figure 3A). Data were quantified by estimating the amount of each E^rns^ protein required to reduce the signal of the uncleaved dsRNA by 50% (Figure 3B; Table 1). E^rns^ of Bungowannah virus was most efficient in degrading dsRNA, being at least as active as the widely studied RNase of BVDV. The enzyme from rodent pestiviruses (Rat77 and Rat99) were also highly active, whereas the E^rns^ proteins of LINDA virus, Aydin-like pestivirus, APPV, and Bat pestivirus were less active, requiring approx. 75 ng/μL for half maximal activity. Finally, E^rns^ of BDV (BD8), pronghorn antelope, and phocoena pestivirus were the least active, being approx. 20- to 30-fold less active than BVDV-E^rns^ (Table 1). The data for the wt E^rns^ proteins from the strains PG2 and H138, both of the species *Pestivirus G*, are not shown, as neither protein showed any dsRNase activity at a concentration up to 200 ng/μL.

In previous studies, we showed that monomeric and dimeric forms of E^rns^ of BVDV were similarly active in degrading dsRNA [44]. Here, we extended this study and investigated the RNase activity of the E^rns^ proteins of Bungowannah and phocoena pestivirus. Exchanging Cys at position 174 and 175 of Bungowannah and phocoena E^rns^, respectively, led to monomeric forms as shown be SDS-PAGE and Coomassie staining (Figure 2B). Surprisingly, monomerization of Bungowannah and phocoena E^rns^ led to a strong decrease and increase in dsRNase activity, respectively, compared to the dimeric form. Similarly, we attempted to artificially induce the dimerization of APPV-E^rns^ by introducing a cysteine residue in either position 182 (APPV-C182-E^rns^) or 190 (APPV-C190-E^rns^), but due to weak Coomassie staining, no clear E^rns^ dimers could be identified (Figure 2B), and no significant change of enzymatic activity could be detected (Figure 3B).

As noted above, the two E^rns^ proteins, expressed either in HEK or CHO cells, from the original strains from the giraffe pestivirus (H138 and PG2; *Pestivirus G*) showed no RNase activity, despite their amino acids located in the enzymatic domain being highly conserved (Figure 1). Similarly, incubation of both E^rns^ proteins with dsRNA at various pH values (i.e., using Tris acetate at pH values of 3.5, 4.5, 5.5, and 6.5, respectively) did not restore any RNase activity. Therefore, we assumed that one or several amino acid changes within a specific region of the protein is responsible for the viral RNase being inactive, even though we did not immediately observe any mutation within the sequence that could obviously be related to a loss of enzymatic activity. Thus, we designed the following three different constructs based on the E^rns^ sequence of the giraffe pestivirus strain H138: (i) “H138-ΔCt”: as BVDV-E^rns^ lacking the C-terminal end (37 amino acids) had a slightly stronger RNase activity than the wild type enzyme [9,24], we expressed H138-E^rns^ similarly lacking the C-terminal end; (ii) “H138-Ncp7-1”: a chimeric protein obtained by substituting the first 71 amino acids of H138 (E1 to L71) with the corresponding ones of BVDV Ncp7-E^rns^; (iii) “H138-Ncp7-2”: a chimeric protein with exchange of the region from amino acids N86 to G148 with the corresponding ones from Ncp7. As the residues between position 71 and 86 are completely conserved among the two giraffe and the BVDV E^rns^ sequences, our constructs were designed upstream and downstream of this region.

The E^rns^ protein H138-Ncp7-1 indeed regained some activity as dsRNase, but only to a low level (the 300 bp stretch of dsRNA could get degraded by 100 ng/μL of E^rns^), whereas the RNase activity of H138-Ncp7-2 -E^rns^ displayed limited RNase activity with only partial dsRNA degradation achieved by 100 ng/μL of E^rns^. On the other hand, H138-ΔCt did not show any RNase activity in the concentration range tested. These results indicated that changes in the N-terminal region of the giraffe pestivirus E^rns^ proteins, rather than in the C-terminus, were responsible to the loss of dsRNA activity.

Finally, the isolation of a pestivirus from cattle in Tanzania belonging to the same genus, *Pestivirus G*, provided the solution to this conundrum. Expression and purification of this protein (labeled as “bovine giraffe pestivirus”, BoGPV) yielded an E^rns^ protein with dsRNase activity comparable to the one from BVDV-Ncp7 or Rat-99 (Figure 3B). Sequence comparison of E^rns^ of BoGPV with PG2 and H138 indicated that the basic amino acid lysine (K) at position 63 of BoGPV (according to the numbering of BVDV-Ncp7; position 64 in Figure 1), which is substituted by the acidic amino acid glutamic acid (E) in PG2 and H138, might play a crucial role in the activity of the protein. Indeed, exchanging the corresponding glutamic acid in PG2 and H138 with a lysine residue completely rescued RNase activity to the level comparable to the one of BVDV Ncp7-E^rns^. Our finding could further be strengthened by the fact that BVDV Ncp7-E^rns^ with the mutation K63E showed a 10-fold reduced activity as dsRNase compared to the wt protein.

### 3.3. Intracellular Localization

**Figure 4:**
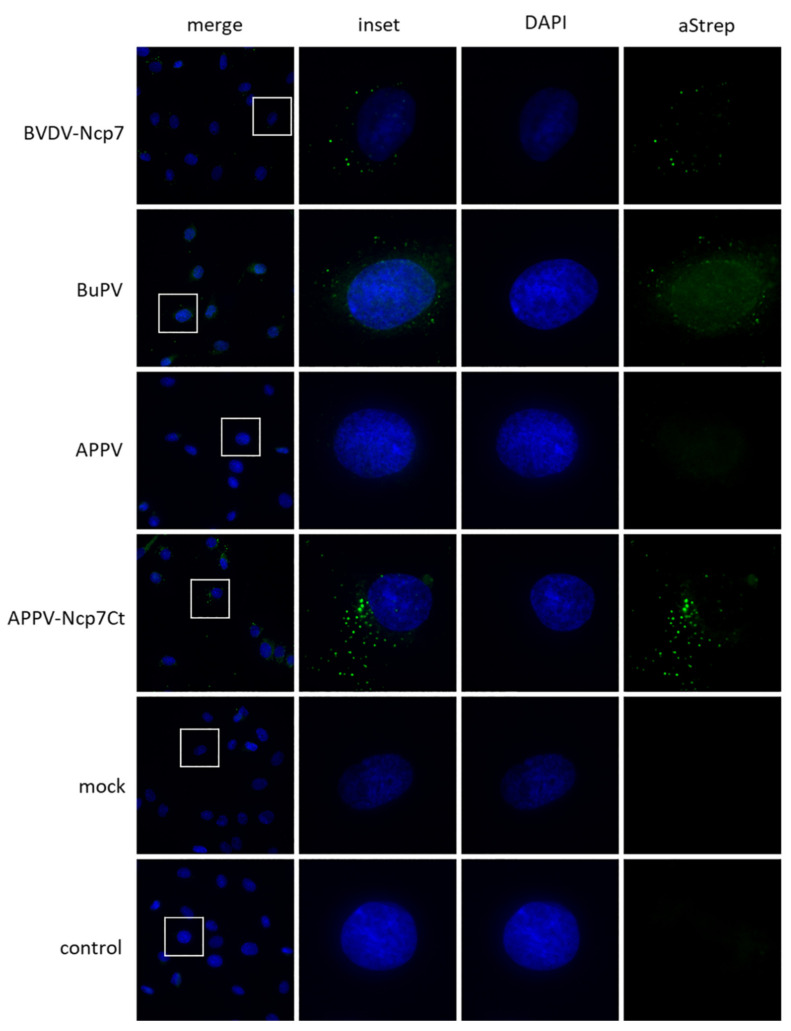
Representative examples of the immunofluorescence (IF) labeling of E^rns^ proteins. E^rns^ was detected by IF microscopy as described using a primary antibody against the Strep-tag present at the C-terminus of each construct and a secondary antibody conjugated with Alexa 488 (**green**). Cells not treated with E^rns^ are labelled as “mock”, whereas cells incubated with BVDV-Ncp7-E^rns^ but treated only with the secondary antibody are labelled with “control”. Nuclei were stained with DAPI (**blue**) present in the mounting medium. The same image processing settings were used for mock and all constructs. For each microphoto, obtained with a 40× air objective, an area was selected (**white square**), magnified five times, and displayed to its right-hand side. One representative experiment out of three is shown. As most of the E^rns^ proteins could not be detected by IF labeling, only BVDV-Ncp7-E^rns^ and BuPV-E^rns^ that gave a clear signal are shown, in addition to one example (APPV-E^rns^) that could not be visualized. By contrast, substituting the C-terminus of APPV-E^rns^ with the one of BVDV-Ncp7-E^rns^ allowed for efficient detection of the chimeric protein.

In previous studies, we provided evidence that E^rns^ must be internalized into its host cell by clathrin-mediated endocytosis in order to act as IFN antagonist [9,24]. Therefore, we investigated the intracellular localization of E^rns^ of the various pestivirus species by immunofluorescence (IF) labeling. A clear IF signal was obtained only for the E^rns^ protein of BVDV and Bungowannah virus (Figure 4). For all the other proteins tested, we could not determine a clear signal by this IF approach. However, chimerizing APPV-E^rns^ with the C-terminus of Ncp7-E^rns^ (termed APPV-Ncp7Ct, containing the last 37 amino acids of BVDV strains Ncp7) rescued the labeling comparable to the one of E^rns^ of BVDV and Bungowannah virus. This suggests that the conformation of the C-terminal amino acids might play a role in the visibility of the Strep-tag for our antibody labeling.

### 3.4. Inhibition of Interferon Expression

**Figure 5:**
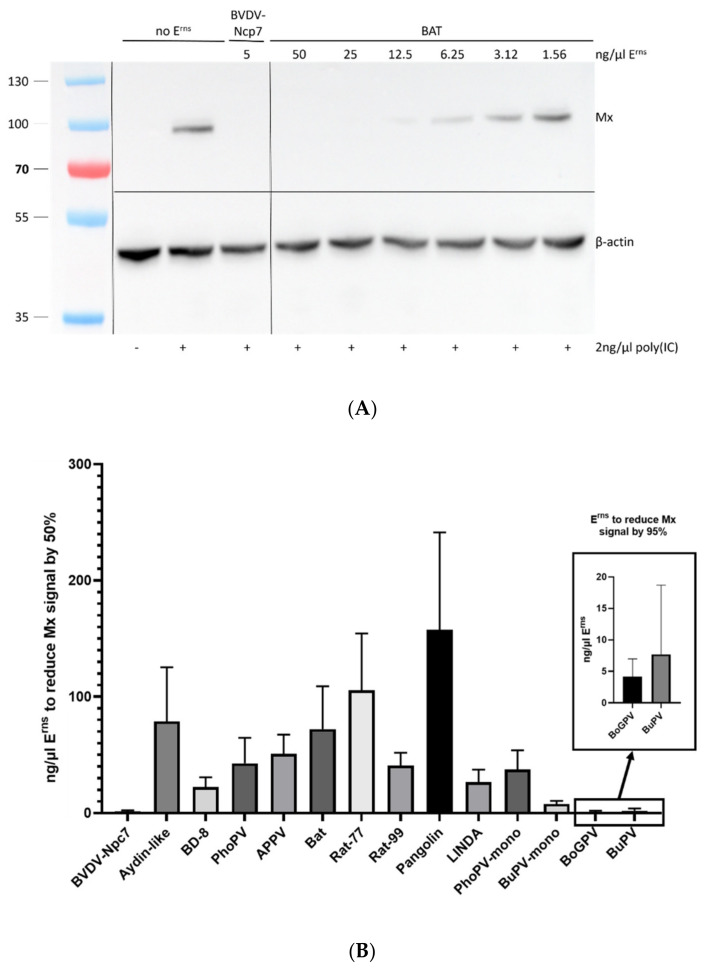
Inhibition of interferon expression was analyzed using the expression of Mx proteins as a proxy. (**A**): Example of a Western blot used to quantify the expression of Mx and the housekeeping protein β-actin in cells incubated with Bat-E^rns^ and subsequently stimulated with poly(IC). The size (kDa) of the pre-stained protein ladder is indicated on the left. The uncropped gel is available in Supplementary Material Figure S3. (**B**): The amount of each E^rns^ protein required to reduce the Mx signal by 50% is reported for the proteins that yielded a signal reduction sufficiently strong to be analyzed (n = 3 ± SD). As BoGPV- and BuPV-E^rns^ were considerably stronger than the other proteins, their results are reported in the inset with a different scale on the y-axis and displaying the amount of these E^rns^ proteins to reduce the Mx signal by 95%. Proteins that did not display any detectable activity (i.e., E^rns^ of Gir-PG2, Gir-H138 and pronghorn antelope) are not shown. The activities of the various E^rns^ proteins in relation to the one of BVDV-Ncp7 is shown in Table 1.

To verify whether the E^rns^ proteins of all the different pestiviruses are able to function as IFN antagonists, at least in cell culture, we pre-treated BT cells for 30 min with a serial dilution of each of the constructs followed by incubation with poly(IC) as synthetic dsRNA for 24 h. Western blots of the cell extracts were performed (Figure 5A), and Mx expression was analyzed and quantified relative to the corresponding β-actin band. To this end, the Mx/β-actin ratio from the well with poly(IC) but without any E^rns^ present in each gel was set to a value of 100% (Figure 5B).

Here, we show that the E^rns^ proteins of all pestivirus species investigated except the pronghorn antelope pestivirus were able to inhibit dsRNA-induced Mx expression, albeit to various extents. E^rns^ of Bungowannah virus was even more efficient than E^rns^ of BVDV, whereas the ones from Aydin-like pestiviruses, Rat77, and Pangolin pestiviruses were least active. For E^rns^ of the strains of the genus *Pestivirus G*, neither strain PG2 nor H138 was able to even reduce poly(IC)-induced IFN synthesis in accordance with their lack of RNase activity. By contrast, the E^rns^ protein of BoGPV, which regained RNase activity, was again able to block induction of the innate immune response by dsRNA with high efficiency (Figure 5B, inset). In addition, we investigated the activity of monomeric forms of Bungowannah- and phocoena-E^rns^ in inhibiting dsRNA-induced Mx expression. Similarly, as reported for monomeric E^rns^ of BVDV-Ncp7 [44], the monomeric forms exhibited similar activity as the corresponding wt form.

## 4. Discussion

Over the last 10 years, the genus *Pestivirus* has seen a great expansion, from four to eleven recognized species [1,52], and with currently eight additional species newly proposed [25]. Typically, pestiviruses encode for N^pro^ and E^rns^, two proteins that are unique to the genus within the flavivirus family, which have pivotal roles in the evasion of the host´s innate immune system [6,7,48]. Specifically, E^rns^ is both, a structural and an enzymatically active protein, which can be found on the surface of the viral envelope or is secreted by the cell as a soluble protein. To date, however, most studies on the role of E^rns^ as IFN antagonist were performed with the enzymes of BVDV or CSFV. Given the central role of E^rns^ in the interaction between the virus and the host and the peculiarities of this protein, we aimed to characterize the E^rns^ proteins from a wide spectrum of pestiviruses whose properties have not yet been characterized.

Here, we provide strong evidence that the envelope glycoprotein E^rns^ of most pestiviruses possess dsRNase activity and is able to block dsRNA-induced IFN synthesis, with only the E^rns^ of pronghorn antelope displaying the lowest activity as RNase and no activity in our in vitro Mx assays. Thus, only the E^rns^ proteins from BVDV-2 (*Pestivirus B*) and ovine pestiviruses (*Pestivirus O*) have not yet formally been shown to be able to act as IFN antagonist, with indirect evidence reported for BVDV-2 [53]. However, based on the sequence conservation and their close proximity to the classical ruminant pestiviruses, it is highly probable that their E^rns^ proteins possess similar activities. This further supports the importance of E^rns^ as envelope glycoprotein and its endoribonuclease activity as IFN antagonist for all viruses classified in the pestivirus genus.

### 4.1. Protein Purification

The various E^rns^ proteins were all expressed as described for E^rns^ of BVDV [44] with a C-terminal extension with a linker region separating the protein from the Strep-tag that was used for affinity purification. During viral replication, E1 is the protein bound to the C-terminal end of E^rns^. The protein cleavage of the E^rns^-E1 precursor is catalyzed by cellular signal peptidases (SP) [43], even though E^rns^ does not have the characteristics of a classical SP substrate with a central hydrophobic, alpha-helical transmembrane domain. Rather, the membrane anchor of E^rns^ is constituted of a C-terminal amphipathic helix laying in plane in the membrane [17,18], followed by a terminal von Heijne cleavage motif [51,54]. To our advantage, the Strep-tag in most of the E^rns^ proteins analyzed in our study, despite their conserved von Heijne sequence, was not cleaved off from their C-terminal end by the SP. This might be due to the facts that truncated E1 peptides are not efficiently cleaved from E^rns^, and that the amino acid sequence of the C-terminal and N-terminal end of E^rns^ and E1, respectively, are additionally involved in recognition by SP [43,50,55].

Nonetheless, we were initially unable to either purify the recombinant protein carrying the wild-type sequence of APPV-E^rns^, or to detect it using an anti-Strep tag antibody. Thus, it might be assumed that the Strep-tag fragment was cleaved away from the construct by the SP. The reason for this difference compared to the other E^rns^ proteins expressed is not known and is not the focus of this study but might well be based on the very divergent sequences, especially of the C-terminal end of APPV-E^rns^, compared to the one of BVDV. To prevent the cleavage of the Strep-tag from APPV-E^rns^, we modified the sequence of the last four amino acids from the von Heijne motive VAEA to GFYA, a change that was shown to inhibit the E^rns^-E1 cleavage in CSFV [43].

### 4.2. RNase Activity

Every E^rns^ protein we initially expressed, with the exception of the two strains of the giraffe pestiviruses, displayed dsRNase activity, but to very different shades (Figure 3). E^rns^ of Bungowannah virus and BoGPV were more effective than E^rns^ of BVDV, whereas the ones from BDV, pronghorn antelope and phocoena pestiviruses were the least effective. By contrast, E^rns^ of H138 and PG2, the two giraffe pestiviruses, were unable to degrade dsRNA and, accordingly, did not inhibit poly(IC)-induced Mx synthesis as marker for the expression of IFN. From their primary sequence, both E^rns^ proteins contain well conserved functional domains, including the RNase domains, the GAG-binding site, and the substrate binding site, which are all similar to the ones described for BVDV-E^rns^ that we used as positive control in our assays. The expression of these E^rns^ proteins in different types of cells or performing the RNase assay at different pH values did not lead to any detectable RNase activity.

This indicated that domains in addition to the ones described are influential in the function of E^rns^ as ribonuclease. To identify the sites that were responsible for the lack of RNase activity, we mutated the E^rns^ protein of the strain H138 by exchanging domains with the corresponding ones from BVDV-Ncp7 by substituting either an N-terminal fragment of H138-E^rns^ (H138-Ncp7-1) or a central fragment (H138-Ncp7-2). Since, in previous experiments, truncation of the C-terminus partially increased RNase activity of BVDV-Ncp7 [9], we also designed a construct with depletion of such region (Giraffe-ΔC). With the exception of Giraffe-ΔC, both chimeric constructs were partially active in degrading dsRNA, with H138-Ncp7-1 and H138-Ncp7-2 being slightly active at 10 ng/µL and 100 ng/µL, respectively, but none of the activities were sufficient to estimate a 50% reduction in the amount of dsRNA. Finally, we expressed the E^rns^ protein according to a new sequence from a giraffe pestivirus that originated from cattle in Tanzania. The amino acid sequence of this protein (labeled as BoGPV) is highly similar to the one of the other two isolates of this species, with 96% similarity to H138 and 95.6% to PG2, respectively. The E^rns^ of BoGPV was highly active in both, the RNase and the Mx assay. A detailed analysis of the amino acid sequence of all three giraffe pestiviruses indicated that the presence of a negatively charged glutamate residue at position 63 in the RNase-inactive proteins compared to a lysine in BoGPV might be the cause. This could clearly be confirmed as the E^rns^ mutants, H138-E63K, and an BVDV-Ncp7-K63E rendered the enzyme active and inactive, respectively, compared to their wt counterparts. These results are corroborated by the observation that according to the structure of the protein, this residue appears to be in proximity of the active site (labeled as Lys 65 in [56]). Thus, the lysine residue residing in a basic patch possibly directs the RNA into the enzymatic pocket, whereas the negatively charged glutamate rather leads to a repulsion of the ribonucleic acid substrate.

We assume that the lack of RNase activity of the E^rns^ proteins of the strains H138 and PG2 is the result of cell culture adaptations. The cytopathogenic strain Giraffe-H138 was isolated in 1967 from a captive giraffe in Kenya ([57] and references therein), and easily replicated in calf testicle and kidney cells and in the MDBK cell line [58,59]. Similarly, the strain PG-2 was isolated by one of us (MS) as cell culture contamination in cells obtained from the Institute of Pathology of our Faculty (hence the name “pathology-giraffe”, PG). The cells were originally used for studies of Theileria infections, and the cells and FBS were originally prepared in Kenya before being moved to Bern. Thus, the precise passage history of the virus isolates is not known, but probably involved a large number of passages in various types of cells. Thus, it can be envisaged that viruses of the species giraffe pestivirus (Pestivirus G) might be widespread in cattle in Africa, and that the isolation from a giraffe might rather have been a spillover event from cattle to giraffe than being a giraffe specific pestivirus per se. This is corroborated by the report of a giraffe pestivirus indeed identified and directly sequenced from cattle in Tanzania. However, the loss of RNase activity by passaging in cell culture appears not to be a general phenomenon, as many BVDV strains, e.g., the NADL strain [60], were passaged for decades in many laboratories without losing their ribonuclease activity.

### 4.3. Intracellular Localization and IFN Antagonism

Most of the proteins analyzed in this study could not be visualized through immunofluorescence labeling targeting the Strep-tag carried on their C-terminal ends, neither by varying the concentration of antibodies used for the labeling nor by increasing the amount of E^rns^ proteins incubated with the cells. It thus might be assumed that conformation of the Strep-tag sequence is not always sufficiently amenable for antibody binding in cell culture. The C-terminus of the proteins clearly has a critical role in the availability of the Strep-tag sequence, as the labeling of APPV- E^rns^ could be rescued when its C-terminus was substituted with the one of BVDV- E^rns^. However, the data of the Mx assay indirectly confirmed that most of these proteins are efficiently transported inside the cells, as an intracellular, possibly endolysosomal localization was reported to be required for the inhibition of the poly(IC)-induced IFN-I cascade [9,24]. Even if we could not use IF to substantiate the level of protein uptake into cells, the lack of a clear correlation between the efficiency of the RNase activity and the ability to inhibit the activation of the IFN system by the various E^rns^ proteins (Table 1) indicate that the effectiveness of entering the host cells might likewise vary between the viral endonucleases of the different pestivirus species. Similarly, we could not determine whether the lack of activity of E^rns^ of the antelope pestivirus in the Mx assay is due to its low RNase activity and/or inefficient or even absent uptake into cells. There is also no obvious variance in its functional domains (Figure 1), and as only the sequence of a single isolate from cell culture supernatant is reported, it remains to be determined whether the E^rns^ protein of antelope pestivirus indeed acts as IFN antagonist.

### 4.4. E^rns^ Dimerization

Our Coomassie staining confirmed the role of the ninth cysteine residue (C171, with respect to the Ncp7-E^rns^ sequence) for the formation of E^rns^ homodimers though intermolecular disulfide linkage [20,61]. All proteins possessing a cysteine in an analogous position were not only assembled as dimers but were also detected as monomers and polymers.

Homodimerization is, in general, an intriguing feature of E^rns^. It has been shown that viruses carrying a monomeric E^rns^ were attenuated in vivo [62], but their replication was not affected in vitro [63]. In contrast to previous interpretations [64], two of the pestiviruses isolated so far, i.e., APPV and Bat pestiviruses, naturally lack homodimeric E^rns^ based on the absence of the ninth cysteine. In our enzymatic assays, the E^rns^ of these two viruses were not performing particularly differently from the other E^rns^ proteins. To further investigate the influence of homodimerization on the in vitro activity of E^rns^, we expressed the E^rns^ of BVDV, Bungowannah and phocoena pestiviruses by substituting the ninth cysteine by an arginine residue. As previously reported, we could confirm that Ncp7-E^rns^ monomer does not report any activity alteration [44], whereas Bungowannah-E^rns^ is impaired in both the RNase activity assay and in the prevention of IFN production in cell culture. By contrast, E^rns^ of phocoena pestivirus recently isolated from harbor porpoises (*Phocoena phocoena*) stranding in the Nordic Sea [38] showed an enhanced enzymatic activity of the monomeric E^rns^ compared to the wild-type construct in both assays. The reason for this difference is unknown. Notably, phocoena pestivirus is the only virus in the pestivirus genus identified to date that lacks the N-terminal autoprotease N^pro^, resulting in a virus with E^rns^ as probably the only IFN antagonist encoded in the genome. In addition, we expressed two mutant forms of APPV with the introduction of an additional Cys at position 182 or 190 (APPV-C182-E^rns^ and APPV-C190-E^rns^) to express a dimeric form of APPV-E^rns^. However, we were unable to clearly identify a shift in the expression of monomeric or dimeric forms compared to the wt proteins, fitting to the unchanged activities as RNase in vitro. As most E^rns^ proteins appear to exist, at least in vitro, in mono-, di- and possibly polymeric forms (Figure 2), the different activities of the various forms might be present and are active concomitantly in an infected host. Currently, there is no clear evidence for an essential difference in function of monomeric versus dimeric E^rns^ in any in vitro assay, except the proposal of an enhanced cell-to-cell spread in BVDV expressing monomeric E^rns^ [65] and, thus, more studies in vitro and in vivo with various mutants of E^rns^ also in the context of infectious virus are required to further clarify the role of E^rns^ in virus replication and evasion of the innate immune response by the large diversity of pestivirus species and in the various hosts.

## 5. Conclusions

In conclusion, this work characterized the main features of E^rns^ from a very broad spectrum of pestiviruses. E^rns^ proteins from the various pestivirus species display largely variable activities (hence the title of the manuscript) as dsRNase and as inhibitors of dsRNA-induced activation of the IFN system in vitro. Thus, every E^rns^ protein expressed by any pestivirus found in nature appears to act as IFN antagonist, with only cell culture adapted strains having lost specific features such as the RNase activity. These results further enhance the basic knowledge to understand the role of E^rns^ as multifunctional viral protein of different members of the genus that co-evolved with their hosts and modulate the reactivity of their innate immune system.

## Figures and Tables

**Table 1:** Summary of E^rns^ of different pestiviruses used in this study. GenBank numbers are only given for wild-type (wt) sequences, and the nomenclature of the pestivirus species was used according to [1,25]. The activities of E^rns^ of BVDV-Ncp7 in the RNase- and Mx-assays (from Figure 3 and Figure 5, respectively) were set to 100%.

Nomenclature Used	GenBank no.	Species	Reference	RNase Assay [%]	Mx Assay [%]
BVDV Ncp7	n.a.	A	[26,27]	=100	=100
BuPV (Bungowannah)	NC_023176	F	[28]	86.24	387.5
APPV	KR011347	K	[29]	6.11	6.07
BD8 (Border Disease)	R4785/06	D	[30]	3.63	13.82
Aydin-like	JX428945	I	[31]	5.75	3.93
Gir-PG2 (Giraffe)	KJ660072	G	[32]	<2.1 ^(^^3)^	<1.55 ^(^^3)^
Gir-H138 (Giraffe)	AF144617	G	[33]	<2.1 ^(^^3)^	<1.55 ^(^^3)^
Pronghorn	AY781152	E	[34]	3.26	<1.55 ^(^^3)^
Rat-77	NC_025677	J	[35]	11.31	2.93
Rat-99	KY370099	Q ^(^^1)^	[36]	22.92	7.59
LINDA	KY436034	L ^(^^1)^	[37]	5.58	11.62
PhoPV (phocoena)	NS170385	M ^(^^1)^	[38]	4.79	7.25
Pangolin	MK636875	P ^(^^1)^	[39]	6.80	1.97
Bat	MH282908	S ^(^^1)^	[36]	6.90	4.29
BoGPV (Giraffe)	n.a.	G	this paper ^(^^2)^	20.76	688.89
BuPV-mono	n.a.	F	this paper	3.91	39.34
PhoPV-mono	n.a.	M ^(^^1)^	this paper	23.91	8.22
APPV-C182	n.a.	K	this paper	6.56	n.d.
APPV-C190	n.a.	K	this paper	7.58	n.d.
H138-Ncp7-1	n.a.	chimera	this paper	2.63	n.d.
H138-Ncp7-2	n.a.	chimera	this paper	<2.1 ^(^^3)^	n.d.
Giraffe ΔC	n.a.	G	this paper	<2.1 ^(^^3)^	n.d.

n.a.: not available; n.d.: not done. ^(^^1)^ Proposed species according to [25]. ^(2)^ Unpublished BoGPV sequence kindly provided by Martin Beer, George Msalya, Silvia Alonzo, and Fred Unger. ^(^^3)^ No activity observed at the highest concentration tested.

## Supplementary Materials

The following are available online at https://www.mdpi.com/article/10.3390/v14020265/s1, Figure S1: Full length uncropped gels for Figure 2; Figure S2: Full length uncropped gel for Figure 3A; Figure S3: Full length uncropped gel for Figure 5A.
