# Supplementary material for: Fifty Shades of Erns: Innate Immune Evasion by the Viral Endonucleases of All Pestivirus Species
Source: Viruses. 2022 Jan 27;14(2):265. doi: 10.3390/v14020265 (PMC8880635; doi:10.3390/v14020265)
Supplement: Supplementary file 1 [file viruses-14-00265-s001.zip › viruses-1551696-supplementary.pdf]

Supplementary Information

Fifty shades of E^{rns}: Innate immune evasion by the viral endonucleases of all pestivirus species

Elena de Martin[1,2,3], and Matthias Schweizer[1,2]

[1] Institute of Virology and Immunology, Laenggass-Str. 122, POB, CH-3001 Bern, Switzerland
[2] Department of Infectious Diseases and Pathobiology, Vetsuisse Faculty, University of Bern, Switzerland.
[3] Graduate School for Cellular and Biomedical Sciences, University of Bern, Switzerland

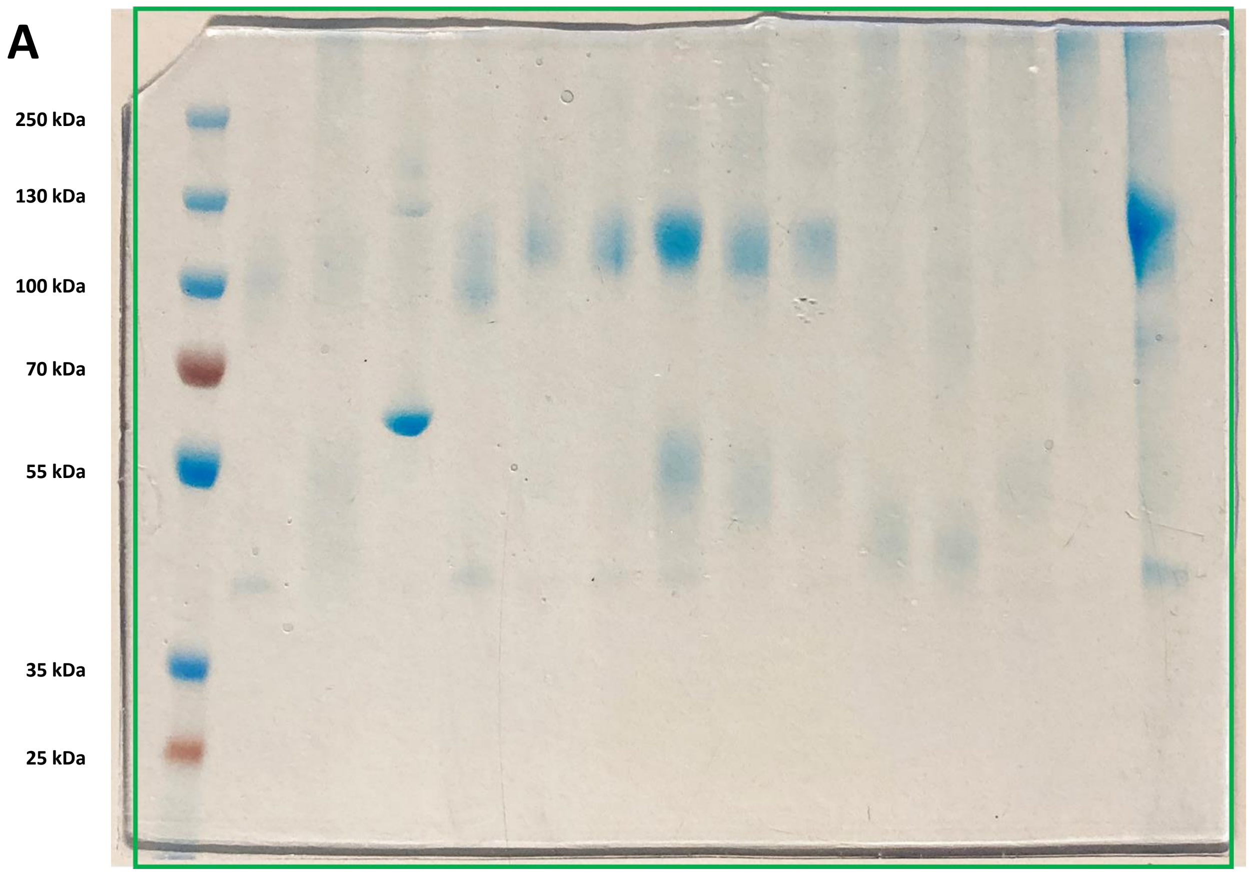

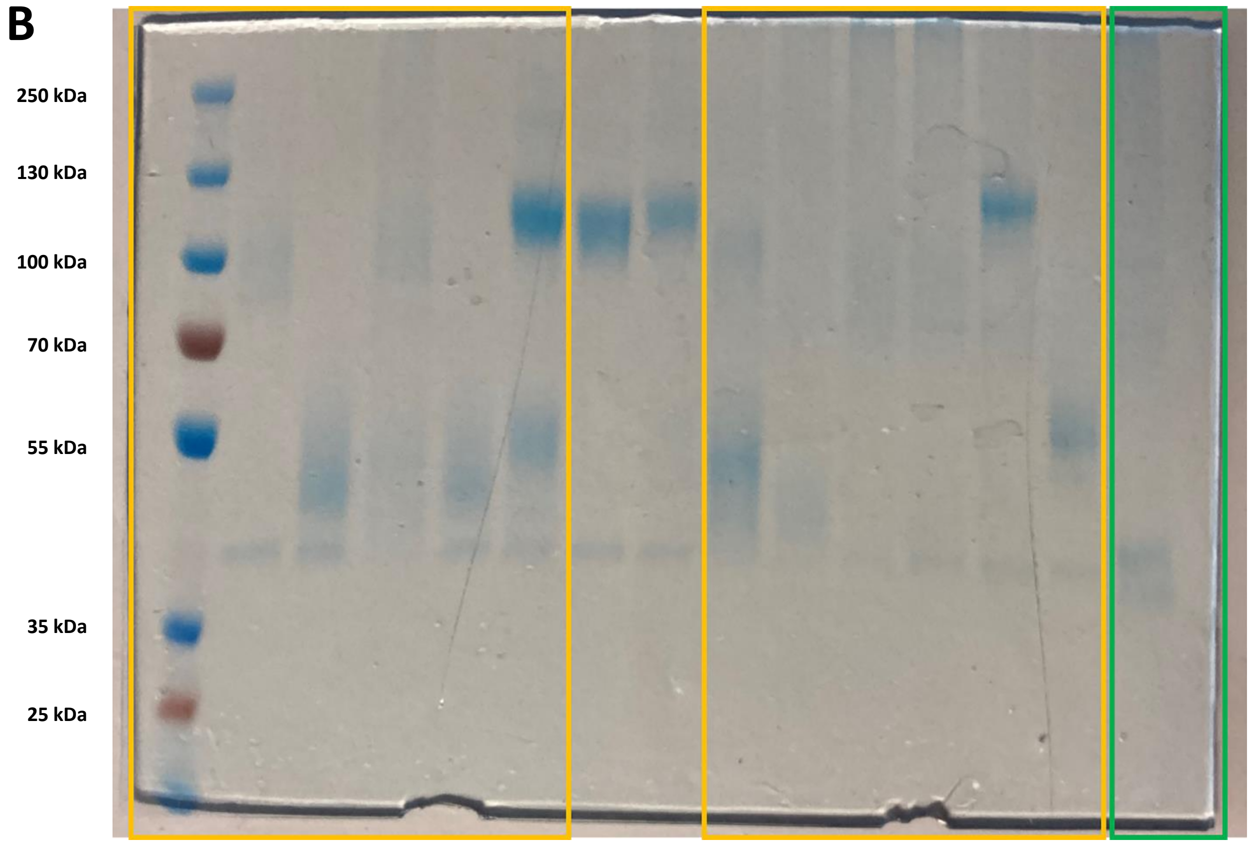

Figure S1. Full-length uncropped gels for Figure 2.
Cropped areas used in Figure 2A and 2B are marked with green and yellow boxes, respectively. The molecular weights for the prestained marker is indicated on the left.

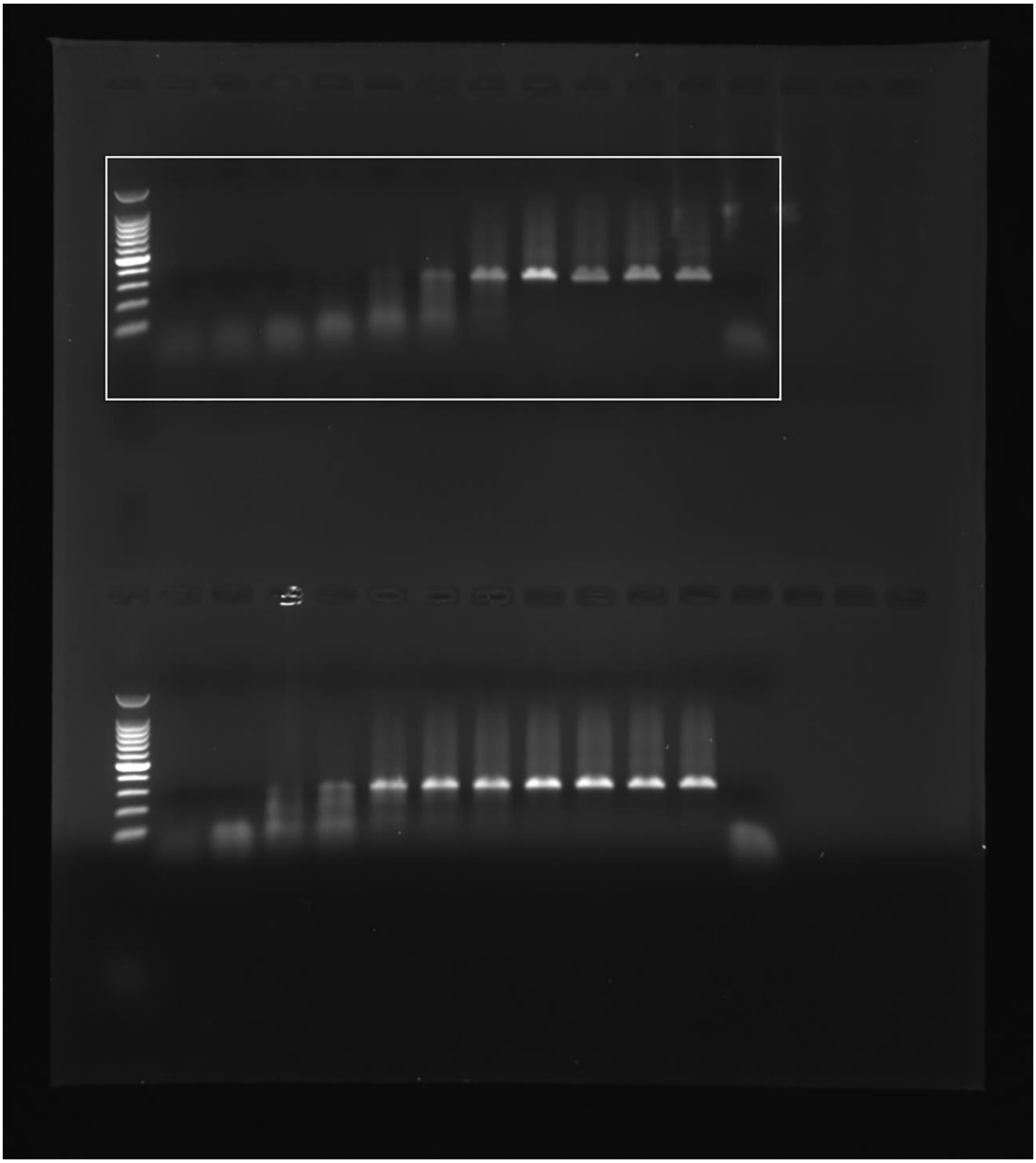

Figure S2. Full-length uncropped gel for figure 3A.
The cropped area used in Figure 3A is marked with a white box. The image used in the article is black/white inverted, and the lower part of the gel originates from another experiment and is not used in the manuscript. The 100 bp DNA size marker is indicated in the left lane, with 500 bp being the brightest band as indicated in Figure 3A.

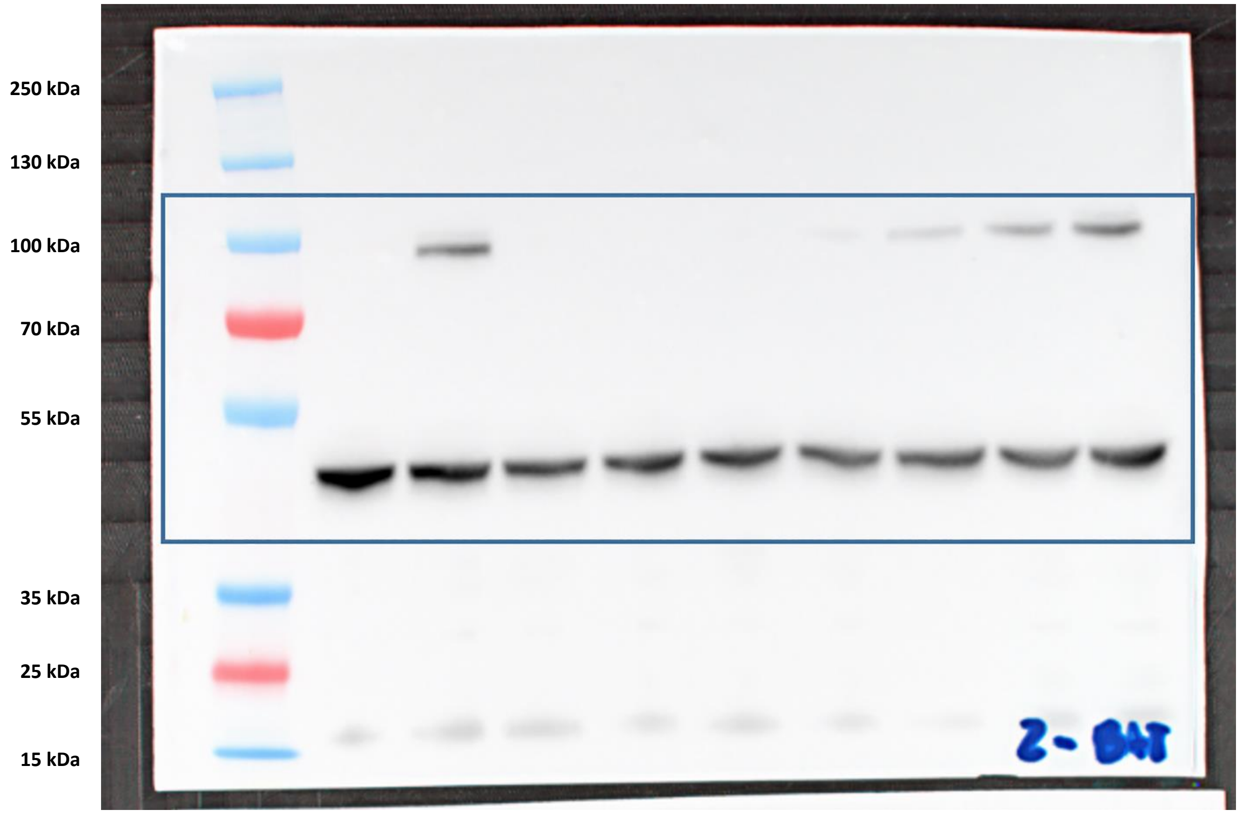

Figure S3. Full-length uncropped gel for figure 5A.
The cropped area used in Figure 5A is marked with a blue box. The molecular weights for the prestained marker is indicated on the left.
